# Parkinson’s disease tremor prediction using EEG data analysis-A preliminary and feasibility study

**DOI:** 10.1186/s12883-023-03468-0

**Published:** 2023-11-24

**Authors:** Sajjad Farashi, Abdolrahman Sarihi, Mahdi Ramezani, Siamak Shahidi, Mehrdokht Mazdeh

**Affiliations:** 1 Neurophysiology Research Center, Hamadan University of Medical Sciences, Hamadan, Iran; 2 Department of Physiology, School of Medicine, Hamadan University of Medical Sciences, Hamadan, Iran; 3 Department of Anatomical Sciences, School of Medicine, Hamadan University of Medical Sciences, Hamadan, Iran; 4 Department of Neurology, School of Medicine, Hamadan University of Medical Sciences, Hamadan, Iran

**Keywords:** Parkinson’s disease, Tremor, EEG, Machine learning

## Abstract

**Purpose:**

Tremor is one of the hallmarks of Parkinson’s disease (PD) that does not respond effectively to conventional medications. In this regard, as a complementary solution, methods such as deep brain stimulation have been proposed. To apply the intervention with minimal side effects, it is necessary to predict tremor initiation. The purpose of the current study was to propose a novel methodology for predicting resting tremors using analysis of EEG time-series.

**Methods:**

A modified algorithm for tremor onset detection from accelerometer data was proposed. Furthermore, a machine learning methodology for predicting PD hand tremors from EEG time-series was proposed. The most discriminative features extracted from EEG data based on statistical analyses and post-hoc tests were used to train the classifier for distinguishing pre-tremor conditions.

**Results:**

Statistical analyses with post-hoc tests showed that features such as form factor and statistical features were the most discriminative features. Furthermore, limited numbers of EEG channels (F3, F7, P4, CP2, FC6, and C4) and EEG bands (Delta and Gamma) were sufficient for an accurate tremor prediction based on EEG data. Based on the selected feature set, a KNN classifier obtained the best pre-tremor prediction performance with an accuracy of 73.67%.

**Conclusion:**

This feasibility study was the first attempt to show the predicting ability of EEG time-series for PD hand tremor prediction. Considering the limitations of this study, future research with longer data, and different brain dynamics are needed for clinical applications.

## Highlights

The potential of EEG for tremor prediction was investigated.

Complexity of EEG time-series in delta band was discriminative for tremor prediction.

Statistical features in gamma band were discriminative for tremor prediction.

EEG biomarkers are suitable for predicting PD resting tremor with accuracy up to 73%.

## Introduction

### Background, aim and objective

Parkinson’s disease (PD) is the second most common neurodegenerative disease worldwide [1]. In the advanced stage of PD, movement disorders such as tremors in the hands, legs, or trunk may appear. Other movement complications such as dyskinesia, bradykinesia, freezing of gaits, and balance problems can also be observed in PD patients [2, 3]. Among these symptoms, tremors respond to conventional medications in a variable manner [4]. Furthermore, tremor is the most visible symptom of PD and reduces significantly the quality of life for such patients. In this regard, several non-pharmacological interventions were proposed for tremor reduction or suppression. Physical activity intervention [5], electrical [6], or magnetic stimulation [7] of muscles or nerves, light therapy [8] or acoustic vibration therapy are among non-invasive methodologies for tremor suppression. Other invasive techniques such as deep brain stimulation (DBS) [9] were also used for tremor suppression in some severe PD cases.

Detecting tremor onset is a critical step in any tremor suppression strategy since applying the intervention in electrical, magnetic, acoustic, or any other form just before tremor initiation reduces the energy consumption of the stimulation device, reduces the total stimulation, and mitigates the side effects such as speech impairments [10] when compared with continuously applied stimulations. As an example, in adaptive deep brain stimulation (aDBS), which is an enhanced version of traditional DBS, delivering stimulation pulses to the deep brain area is controlled by motor symptoms such as tremor initiation [11]. For suppressing PD tremors through external intervention, the initial step is to detect the onset of tremors. Different methodologies were proposed for detecting PD tremors. Some methods focused on data collected by accelerometer and gyro sensors attached to the body surface [12–16]. Some others utilized electromyography (EMG) data [17], while other methods were focused on the neural data acquired from brain [18–22]. The latter methods typically use data from electrodes implanted in deep brain areas through an invasive procedure.

In this study, we aimed to test the feasibility of predicting hand tremors using dynamical changes in electroencephalography (EEG) rather than relying on data obtained from attached accelerometer sensors, EMG, or implanted electrodes. EEG reflects the real-time manifestation of numerous motor and psychological functions, making it suitable for the automatic detection of such functions. In the case of tremor suppression based on accelerometers or EMG sensors, first, the tremors should initiate, recorded data be processed and hand movement characteristics satisfy tremor-specific characteristics, then the intervention be applied. This introduces a delay between the tremor onset and intervention that reduces the effectiveness of tremor suppression. Furthermore, invasive methodologies for tremor suppression, such as DBS, may cause complications such as the risk of intracranial hemorrhage [23]. EEG signals can be recorded non-invasively, and because they originate from the nervous system, we hypothesized that they can provide useful information for predicting tremors before tremor manifestation.

### Related works

EEG signals were successfully utilized for the diagnosis of PD patients from healthy subjects [24, 25]. Furthermore, EEG signals were used to characterize the electrical activities of the brain in PD patients and to highlight the differences with healthy individuals [26, 27]. Such studies assisted researchers to investigate the neural mechanisms underlying PD. For predicting movements, EEG signals were used extensively. Movement-related cortical potentials (MRCP) are positive or negative deflections in EEG data that appear just before the onset of intentional movements. The Bereitschaftspotential component of MRCP was proposed as a biomarker for the prediction of movement intention [28]. The time-frequency characteristics of EEG data were used to predict different hand movements with moderate accuracy [29]. Single-trial EEG signals were also employed for the prediction of hand movement speed and force [30].

While strategies such as analysis of local field potentials recorded by deep brain implanted electrodes [16, 21, 22], assessing hand movement using inertial sensors [15, 31], or analysis of hand movement with electromyography [17] were proposed for predicting PD tremor, to the best of the authors’ knowledge, there is no study using EEG signals to predict tremor onset in PD patients. Therefore, the current study focused on the feasibility of using EEG signals to predict resting hand tremors in such patients. Additionally, it was also of special interest to check if limited number of EEG recording channels could achieve predictive capabilities.

## Materials and methods

### Dataset description

#### EEG dataset

The association between EEG dynamics and tremors requires a dataset that include simultaneous recording of EEG and hand movement data. To address this, a publicly available dataset provided by the University of New Mexico was utilized in which synchronized EEG and hand movement data were accessible (available from: http://predict.cs.unm.edu/downloads.php under the Parkinson’s Rests project). This is a multimodal dataset including multi-channel EEG data, 3-axis accelerometer data (in the x, y, and z direction) and vertical eye movement profiles. Twenty-seven PD cases in two conditions (ON medication: continuation of dopaminergic medication; OFF-medication: withdrawal of dopaminergic medications 15 h prior to recording) visited the lab in a counterbalanced manner across ON and OFF-medication conditions. Two participants were excluded due to the lack of hand movement data. The time interval between data recording sessions for the ON and OFF-medication conditions was one week. This dataset also includes data from 25 age- and sex-matched healthy participants. In the PD group, participants underwent a battery of behavioral tests, including Mini Mental State Exam (MMSE), Beck Depression Inventory (BDI) test, North American Adult Reading Test (NAART), and Unified Parkinson’s Disease Rating Scale (UPDRS) test (for more details, refer to [32]). All included participants had enough high cognitive score  (MMSE > 26). EEG data were recorded using a 64-channel EEG recorder in accordance with the 10–20 standard and digitized at a sampling rate of 500 Hz. For two resting-state brain conditions, eyes-open and eyes-closed, 1-minute data was recorded, simultaneously vertical eye movements data using EMG electrode and hand motions using a 3-axis accelerometer was captured [33].

#### Accelerometer data

In the current study, a modified algorithm for automatic tremor detection from accelerometer data of PD patients was proposed. To evaluate the performance of the tremor detection algorithm, it was essential to have a ground truth data where tremor onsets were accurately labeled. For this purpose, the hand movement data labeled with tremor onset time from three PD patients was recorded using an IMU sensor (BWT901CL, Wit motion, China). The IMU sensor was attached to the most affected hand of each PD patient. Using a customized code that was developed based on the provided source codes and sensor driver (available at: https://github.com/WITMOTION), tremor data was recorded during the hand resting state. During recording, each participant was instructed to sit in a relaxed position in a chair with both hands on the armrest. An expert supervisor identified tremor onset and end time during recording by pressing prespecified keys on the computer keyboard, guided by visual inspection. The sensor’s sampling frequency was set to 100 Hz, and the recording duration was 5 min. If the handshake persisted for more than 30s, the supervisor asked the participant to perform a voluntary movement in order to terminate the resting tremor. This was done for increasing the number of tremors for statistical analysis purposes. Data from a 3-axis accelerometer (in x, y, and z directions) and the tremor onset time for each subject were recorded in an Excel file for further analysis. Real tremor onset times and the detected tremor times were compared to assess the performance of the proposed tremor detection algorithm.

**Fig. 1:**
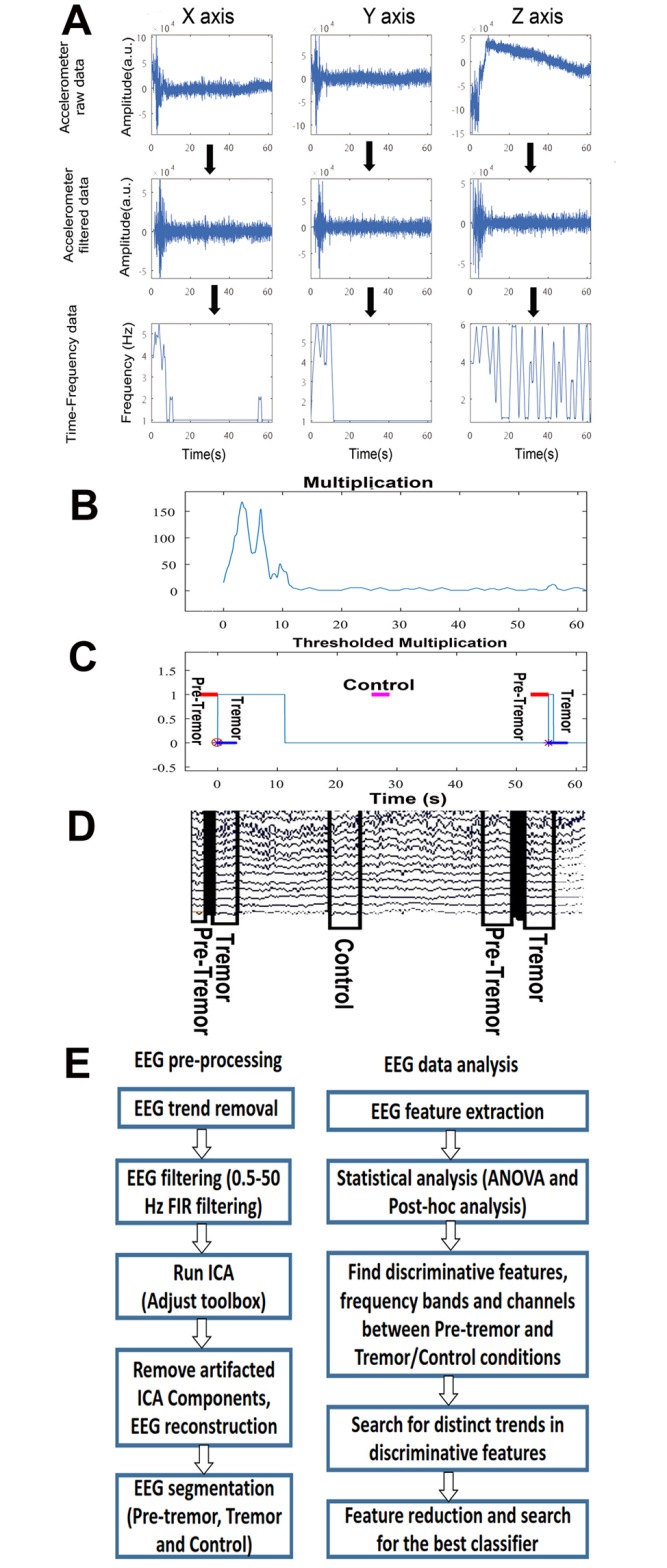
Block diagram of the proposed methodology. Accelerometer raw data (panel A, upper), filtered accelerometer data (panel **A**, middle) and its time-frequency profile (panel A, lower). Multiplication of time-frequency profile of x, y and z-axis accelerometer data (**B**). According to a thresholding strategy, pre-tremor, tremor and non-tremor (control) segments were detected (**C**). Based on the onset time of tremor, pre-tremor and non-tremor segments, related EEG segments were extracted by windowing (**D**). Preprocessing and analyses blocks (**E**)

The block diagram of the proposed methodology is shown in Fig. 1. In summary, the hand accelerometer data in three directions ( x, y and z) were filtered and time-frequency representation was calculated. To account for hand vibrations in all directions, the multiplication of accelerometer data across the three directions ( x, y and z) was calculated. Using a thresholding strategy, tremor onset and endpoint times were found. After preprocessing the EEG data (including trend removal, filtering, and artifact removal), tremor onset time and tremor endpoint time were used for extracting three segments or epochs (pre-tremor, tremor, and non-tremor segments) from the EEG time series. Notably, EEG time series and accelerometer data were recorded simultaneously. From each EEG extracted epoch, several features were extracted and the best discriminative sets were used for tremor prediction. More details can be found in sections  2–2, 2–3, and 2–4.

### Tremor onset detection algorithm

**Fig. 2:**
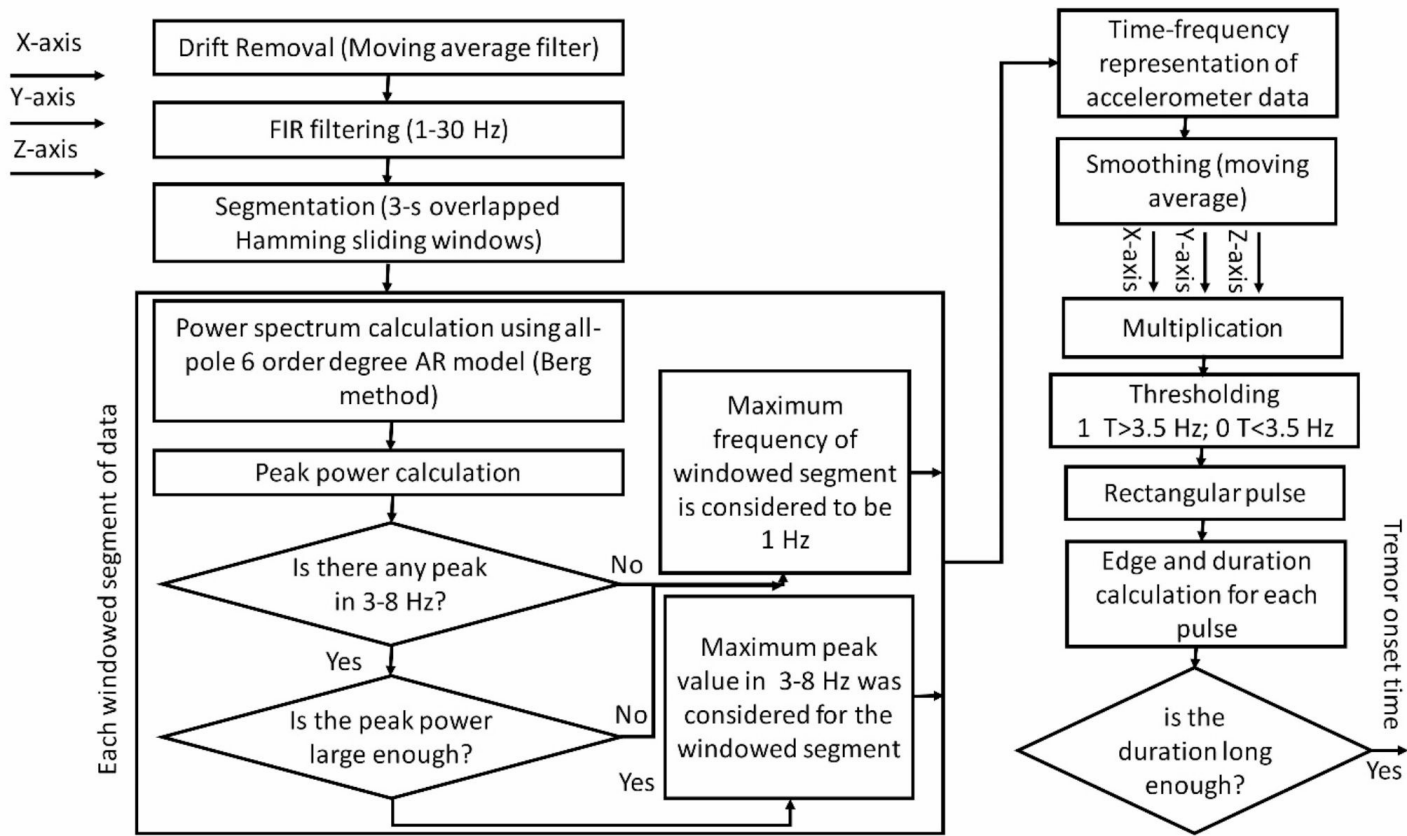
Block diagram of the proposed tremor-onset detection algorithm

For tremor detection, we used the modified version of the algorithm proposed by Salarian et al. [16]. The accelerometer data in each axis (x, y, and z) was analyzed separately. The following steps were performed consecutively: (1) Drift of each channel was removed using a moving average filter. (2) To eliminate very slow and fast non-PD tremor fluctuations, the accelerometer data was filtered using a bandpass finite impulse response (FIR) filter with cut-off frequencies of 1 and 30 Hz [14]. (3) The filtered data was segmented using 3-second sliding overlapped Hamming windows (90% overlap ratio) to emphasize the samples in the center of the window. (4) For each window, an all-pole sixth-degree autoregressive model using the Berg method calculated the frequency spectrum of the signal. The peak power frequencies of the calculated spectrum were found. If the spectrum exhibited peak powers in 3–8 Hz range (slightly wider than the frequency range of PD tremors), the window was labeled as a tremor window and the maximum peak frequency of the windowed data within the 3–8 Hz span was considered for that window. Otherwise, the windowed segment was labeled as non-tremor and the frequency of that segment was considered to be 1. The reason for allocating the value of one (rather than zero) is that in the final stage, the processed accelerometer data of the x, y, and z axes were multiplied. Tremors are heterogeneous events that appear in different directions (i.e. x, y, and z axes). Therefore, combining tremor detection across multiple axes using multiplication and assigning a value of 1, even when tremor isn’t observed in all axes, helps preserve the tremor profile in the overall calculation. (5) To avoid capturing very low-amplitude hand vibrations, the tremor-labeled window needed to exhibit a peak frequency greater than T, where T is a threshold calculated by dividing the maximum peak power of accelerometer data by 10. (6) The values of dominant peaks from successive overlapping windowed segments were obtained. To estimate the start and endpoint of the tremor event, the result of the above procedure was upsampled to match the length of the original accelerometer data and time series. This resulted in a time-frequency representation of the accelerometer data across the x, y, and z axes. (7) To eliminate the effect of unwanted transient perturbations in the upsampled data, it was smoothed. (8) Since the tremors can occur in any direction (x, y, and z-axis), to preserve the tremor information, the combination of all three axes was considered [16]. In contrast to Salarian et al., who summed up the results for accelerometer data from x, y, and z axes, we multiplied the profiles for different axes. This multiplication approach highlighted the tremor event compared to summation. (9) Since PD resting tremor frequency is mainly concentrated in 3.5–7.5 Hz range [16], in case of the presence of hand tremors in at least one axis, the multiplication of time-frequency profiles should be larger than 3.5 Hz. A threshold was applied to the multiplication and a rectangular pulse waveform was obtained which contained rectangular pulses according to the number of dominant tremors. The edge and duration of rectangular pulses were used for detecting tremors and the duration of each tremor. The tremor was acceptable if the hand vibration was non-transient and persisted for more than 3 s. The procedure outlined above identifies dominant resting-state PD tremors. The block diagram for tremor-onset detection algorithm is presented in Fig. 2.

### EEG data preprocessing

For EEG data preprocessing, the baseline of each channel was removed using a moving average filter. A linear phase FIR filter was used to remove unwanted fluctuations outside the frequency range of classical EEG waves (i.e. 0.5–50 Hz). Using the linear phase filter prevents the phase distortion of data. In order to remove non-EEG fluctuations with the overlapped frequency content with EEG (for example EMG or eye movement patterns), independent component analysis (ICA) was employed using ADJUST plugin (version 1.1.1). The infected ICA components were removed and the pure data was reconstructed using the remaining ICA components. EEG data were segmented for eyes-open and eyes-closed conditions according to the provided data labels.

### Feature extraction

By detecting tremor onset, a 3-second segment of EEG data before tremor onset and 3-second of data after tremor onset were extracted as ‘Pre-tremor’ and ‘Tremor’ segments for further analysis. Additionally, a non-overlapping 3-second segment of data with the ‘Pre-tremor’ and ‘Tremor’ segments was extracted and labeled as the ‘Control’ or ‘non-tremor’ segment for statistical analyses. For each segment of EEG data, various types of features were extracted. These features are described in Table 1. Extracted features were related to both time and frequency domain characteristics of the EEG time-series (63 channels of EEG data and 25 patients) as well as entropy as a useful measure of information retrieval in EEG time-series [34]. The feature set was extracted for two different brain resting-state conditions (i.e. eyes-closed and eyes-open) and two conditions of OFF- and ON-medication states.

**Table 1:** Extracted features from EEG time-series (X) for tremor prediction

Feature name	Category	Description
Entropy(E)	Time-domain	Shannon entropy for wavelet coefficients in each decomposition node of wavelet packet. The final entropy is calculated by the summation of entropy values for all nodes of wavelet packet. $E=\sum _{i}E\left({s}_{i}\right)$ Where s_i_ is the vector of wavelet coefficients in *i-th* node and E is the entropy. $E\left({s}_{i}\right)={{s}_{i}}^{2}log{{s}_{i}}^{2}$
L-moments (L-scale, L-skewness, L-kurtosis)	Time-domain	L-moment are statistics calculated by linear combination of conventional moments [35]. L-moments are more robust against outliers compared with conventional moments.
Form Factor (FF)	Time-domain	The ratio of the mobility of the first derivative of the signal to the mobility of the signal [36], where mobility is the ratio of standard deviation for first derivative of time-series and the time-series itself: $FF=\frac{{\sigma }_{{X}^{{\prime }{\prime }}}/{\sigma }_{{X}^{{\prime }}}}{{\sigma }_{{X}^{{\prime }}}/{\sigma }_{X}}$
Sample Entropy (Sen)	Time-domain	Negative logarithm of conditional probability of the successive segmented time-series samples. It is an indicator of time-series complexity [37]
Root mean square (RMS)	Time-domain	${X}_{RMS}=\sqrt{\frac{1}{N}\sum _{n=1}^{N}{\left|{X}_{n}\right|}^{2}}$
Conventional statistics (median, mean, variance, skewness, kurtosis, higher order statistics (5th and 6th momentums)	Time-domain	The mean value, median value, variance, kurtosis, skewness and 5th and 6th order statistics. These statistics are dependent to the distribution of data points of time-series.
Peak frequency (Hz)	Frequency-domain	The frequency in which maximum value of power spectral density was observed.
Band power (Hz)	Frequency-domain	The average power in the input signal.
Power band width (Hz)	frequency-domain	3dB bandwidth (half power bandwidth). Using the peridogram power spectrum estimate by a rectangular window, the frequency difference between points in which the spectrum is at least 3dB lower than the maximum point of spectrum.

### Statistical analysis for feature selection

The normality distribution of each feature among patients was checked using the Lilliefors test [38] with a significance level of 0.05. This two-sided test checks the goodness-of-fit to the normal distribution for samples from an unknown distribution. To check if each extracted feature could distinguish pre-tremor condition from tremor and non-tremor control conditions, in the case of normal distribution of a feature, one-way analysis of variance (ANOVA) was used, while for non-normal distributed features, Kruskal-Wallis non-parametric test was used. These tests assessed whether the mean or median values of a feature among our three groups (i.e. ‘Pre-tremor’, ‘Tremor’, and ‘Non-tremor control’) were significantly different. To identify specific difference between the ‘Pre-tremor’ group and two other groups, the post-hoc multiple-comparison test was conducted using the Tukey-Kramer method to the obtained p-values. In order to select the most discriminative features, the significant level was adjusted to 0.01. Statistical analyses were conducted using MATLAB 2017a and its Statistical toolbox.

### Classification strategy

The classification step aimed to assess the performance of the most discriminative features in distinguishing between pre-tremor and tremor/control conditions. The extracted and selected features were fed to different types of classifiers, including k-nearest neighbor (KNN), decision tree, multi-class support vector machine (SVM), Naïve Bayes, and discriminant analysis. For the multi-class SVM classifier, an error-correcting output codes (ECOC) model with a one-versus-all coding design was used that changed the multi-class classification problem to a set of binary classifications [39]. The hyperparameters for SVM were RBF kernel function, an Iterative Single Data Algorithm as optimization routine, a box constrain (parameter for controlling the maximum penalty on margin-violating observations) of 10, and a gamma parameter (kernel scale parameter) of 1. For the multi-class KNN classifier (k = 3 neighbors), the nearest neighbors were found using an exhaustive search algorithm and Minkowski distance. The automatic parameter optimization for KNN classifiers was used by the built-in functions of MATLAB. For decision tree classifier, Maximum tree depth was set to 10, maximal number of decision splits was set to 5, and a Gini’s diversity index as splitting criterion was used. For the Naïve Bayes classifier, a Gaussian kernel smoothing type, and an unbounded Kernel smoothing density support were used. Since features were labeled as “Pre-tremor”, “Tremor” and “Control”, the classification procedure was a kind of supervised classification. The classification performance was evaluated using sensitivity (SE), specificity (SP), and accuracy (ACC) calculated with the following formula:

1 $$SE=\frac{TP}{TP+FN}$$

2 $$SP=\frac{TN}{TN+FP}$$

3 $$ACC=\frac{TP+TN}{TP+TN+FP+FN}$$

In Eqs. (1–3), *TP*, *FN*, *TN*, and *FP* represent true positive, false negative, true negative, and false positive, respectively. To check the sensitivity of the performance metrics to the input feature space, K-fold cross-validation was used. Furthermore, area under the receiver operating curve (*AUC*) was calculated for each classifier. The classification was performed using “Statistics and Machine Learning toolbox” of MATLAB 2017a.

## Results

### Tremor detection from accelerometer data

The results of the proposed modified tremor detection algorithm are reported in Table 2. These results were obtained according to the hand tremor data recorded from 3 PD patients in 5 min recording duration using a 3-axis accelerometer. In order to investigate the effect of non-tremor events on the performance of the proposed algorithm, the results were reported for both low and high SNR accelerometer data. For the low SNR data, the patient was instructed to perform some voluntary hand motions when the hand was on the arm of the chair. In contrast, for high SNR data, participant was asked to avoid voluntary movement. In Table 2, performance was evaluated according to accuracy and false alarm rate. Accuracy measures the proximity of detected tremor onset time to the true value (100 ms threshold) and false alarm rate shows the probability of incorrect tremor detection.

It should be noted that for some patients, the accelerometer data indicated continuous hand tremor throughout EEG recording. Furthermore, for a few patients, no tremor was observed during EEG recording in eyes-closed or eyes-open conditions. These patients were excluded for the analyses because it was not possible to identify the pre-tremor or tremor segments.

**Table 2:** Performance of the proposed tremor detection algorithm

	Accuracy (%)	False alarm rate (%)
High SNR	97.22 ± 0.23	1.28 ± 0.2
Low SNR	89.17 ± 2.30	4.33 ± 1.78

### Discriminative features

The results in Table 2 indicated that the modified tremor detection algorithm achieved acceptable accuracy and a relatively low false alarm rate. Table 3 showed the features, frequency bands, and EEG recording locations where pre-tremor status was distinguished from tremor and no-tremor conditions (multiple comparisons corrected, p < 0.01). These results were obtained under different conditions including eyes-closed, eyes-open, ON-medication, and OFF-medications.

**Table 3:** Features that discriminate Pre-tremor condition from Tremor and Control conditions (multiple comparisons corrected p-value < 0.01)

Condition	Feature	EEG frequency band	EEG channel	Description
**Off-medication, eyes-closed**	Form factor	Delta	F7, FC5, FC1, PZ, O1, P4, P8, CP6, CP2, C4, CP3, P1, P5, PO3, POZ, PO8, P2, CP4, TP8, FT8	The feature value for pre-tremor is significantly higher compared with tremor and control conditions (p < 0.01) in 3-s window.
	Peak frequency	Gamma	FC6	
**ON-medication, eyes-closed**	Form factor	Delta	F4, P4	Significantly larger value for 3-s pre-tremor segment compared with the same duration of tremor and control conditions (p < 0.01).
		Alpha	C3, CP5, CP1, CP2, CP3	
	HOS5	Gamma	F3	
	Skewness	Gamma	F3	
	Kurtosis	Gamma	FC2, FT7, FCZ	
	HOS6	Gamma	FC5, FC6, FC2, FT7, FCZ	
	L-skewness	Gamma	F7	
	L-kurtosis	Gamma	C4, FT10	
**ON-medication, eyes-open**	Form factor	Theta	C2	Significantly larger value for 3-s pre-tremor segment compared with the same duration of tremor and control conditions (p < 0.01).
		Alpha	CP2	
	HOS5	Gamma	FZ, F3, F5	
	HOS6	Gamma	FC5, FC6, FC2, F1, FC3, FT7, FCZ	
	Kurtosis	Gamma	FC6, FC3, FT7, P1	
	Skewness	Gamma	F3, F5	
**OFF-medication, eyes-open**	Form factor	Delta	FZ, F3, F7, FC5, C3, TP9, CP5, CP1, PZ, P3, O1, O2, P4, P8, CP6, CP2, CZ, C4, FC2, F1, TP7, P1, P5, PO3, POZ, PO4, PO8, P6, P2, CP4, TP8, C6, C2, F2, AF4	Features for pre-tremor value are significantly larger compared with tremor and control conditions (p < 0.01) in 3-s window.

According to the results reported by Table 3, EEG features, frequency bands, and channel locations in which pre-tremor condition was significantly differed from tremor and control conditions were specified (p < 0.01). However, in real situations there is no label on EEG data for pre-tremor or tremor conditions; therefore, it is important to find a way to relate discriminative features to the tremor events. One hypothesis was that the temporal change of features during time-evolution might provide an alert for tremor onset prediction. To investigate this hypothesis, EEG data in a 3-second window before tremor onset, after tremor onset and during the control condition were segmented to 500ms overlapped subsections (98% overlap ratio) and the feature was recalculated in successive windowed segments. This obtained the time-evolution of the feature during pre-tremor, tremor, and control conditions with the aim of identifying a distinctive pattern between pre-tremor, tremor and control conditions. In this regard, the feature can be used for tremor prediction. According to this analysis, no clear pattern for the temporal change of none of the features during the pre-tremor condition was observed. As an alternative strategy, constructing a classifier using the most discriminative features (refer to Table 3) to differentiate between the three conditions (i.e. Pre-tremor, Tremor, and Control) was considered. Such a trained classifier could be employed for real-time tremor prediction. As Table 3 showed, the feature sets were different across different brain dynamics (i.e. resting-state eyes-open or eyes-closed conditions and ON-medication or OFF-medication conditions). For classification, features and frequency bands for different dynamics obtained by Table 3 were accumulated (feature set No.1), focusing on restricting the number of EEG channels. For example, the form factor feature in the delta-band calculated from P4 location was discriminative for OFF-medication eyes-closed, OFF-medication eyes-open and ON-medication eyes-closed conditions. In this regard, the form factor in the delta-band calculated from P4 channel was selected as a feature for classification, even though it was not discriminative for the ON-medication eyes-open condition. In this manner, the following feature set (feature set No.1) were selected for classification: ([Form factor, delta, P4],[ Peak frequency, Gamma, FC6], [Form factor, Alpha, CP2], [HOS5, Gamma, F3], [Skewness, Gamma, F3], [Kurtosis, Gamma, FC6], [HOS6, Gamma, FC6], [L-skewness, Gamma, F7], [L-kurtosis, Gamma, C4]). To explore the possibility of reducing the number of features (and EEG channels) while maintaining acceptable classification results, the neighborhood component analysis (NCA) was also performed. NCA reduced the Feature set No.1 to Feature set No.2. It was observed that feature set No.2 varied across different runs of the algorithm based on the training and test sets; therefore, it was not possible to report Feature set No.2 in a unique manner.

### Classification results

Results for classification were reported in Table 4. The input of the classifiers included different brain dynamics including eyes-open, eyes-closed resting states, ON and OFF-medication states.

**Table 4:** Classification results for tremor prediction applying different feature sets and classifiers. The results were mean ± std for a 10-fold cross validation

Feature set	Classifier	Accuracy (%)	Sensitivity			Specificity			AUC
Feature set No.1	KNN	**73.67 ± 9.56**	**70.90 ± 14.89**	**87.18 ± 10.00**	**61.52 ± 14.53**	**90.54 ± 8.92**	**92.50 ± 6.10**	**79.4 ± 9.30**	**0.74 ± 0.09**
	Multi-class SVM	45.44 ± 7.96	42.31 ± 30.09	67.37 ± 25.56	20.57 ± 23.33	72.19 ± 11.93	65.8 ± 35.3	74.4 ± 16.31	0.45 ± 0.08
	Decision tree	63.62 ± 8.32	55.25 ± 17.38	82.22 ± 15.40	61.92 ± 14.46	83.81 ± 12.62	89.74 ± 5.25	72.66 ± 12.27	0.64 ± 0.08
	Nave Bayes	61.85 ± 13.25	74.68 ± 30.19	74.37 ± 28.23	63.63 ± 38.91	89.62 ± 8.44	92.76 ± 6.54	72.53 ± 16.50	0.62 ± 0.13
	Discriminant analysis	54.78 ± 5.85	59.67 ± 31.05	85.65 ± 15.10	41.78 ± 13.10	70.17 ± 8.11	80.01 ± 8.11	90.23 ± 11.31	0.55 ± 0.06
Feature set No.2	KNN	**81.29 ± 9.19**	**79.15 ± 11.38**	**84.86 ± 11.53**	**83.94 ± 20.93**	**90.37 ± 10.33**	**95.62 ± 6.29**	**86.57 ± 4.50**	**0.81 ± 0.09**
	Multi-class SVM	51.64 ± 8.97	51.71 ± 30.99	64.55 ± 24.15	23.10 ± 29.89	71.68 ± 12.46	88.17 ± 7.71	78.48 ± 15.71	0.52 ± 0.09
	Decision tree	65.96 ± 4.41	66.85 ± 6.62	72.95 ± 13.55	59.93 ± 17.38	86.57 ± 5.05	82.72 ± 4.83	79.62 ± 5.44	0.66 ± 0.04
	Nave Bayes	71.49 ± 11.44	85.98 ± 14.09	76.62 ± 22.58	67.84 ± 19.76	82.81 ± 5.98	91.67 ± 8.55	86.74 ± 12.66	0.71 ± 0.11
	Discriminant analysis	56.32 ± 3.69	81.67 ± 21.08	71.31 ± 10.16	47.63 ± 6.02	67.51 ± 7.08	87.06 ± 8.16	88.85 ± 10.69	0.56 ± 0.04

### Effect of window size on the results

In a pilot study, very short (1 s), medium (4 s) and very long (10 s) segments were tested. However, the results indicated that very short or very long segments were not good choices for our analysis in terms of classification accuracy or real-time feasibility. It should be noted that for a short length window, the calculation of features such as sample entropy or features extracted from power spectrum might not be reliable. Furthermore, for a longer window length, the computational and time complexity might increase. This degrades the real-time feasibility of the algorithm. For medium length EEG segments, two different length of segments (3 and 5 s segments) were also tested. In Table 5, the effect of window size on the classification result was reported. Comparing 3 and 5 s windows, it was observed that for 5 s window, none of the features were discriminative for eyes-open and eyes-closed ON-medication conditions. For 5s windows, only ‘Form factor’ feature in the delta band was discriminative (at C3, P6, PO4, P3, PZ and CP1 for eyes-open and at PZ, CP1, C3, and C3 for eyes-closed condition, respectively). In Table 5, the best classification performance of the proposed methodology for pre-tremor prediction was compared for 3 and 5 s windows. For a 5s window, the discriminative features for all dynamics (i.e. eyes-open, eyes-closed; ON-medication and OFF-medication) were [Form factor, Delta, C3];[Form factor, Delta, CP1];[Form factor, Delta, Pz];[Form factor, Delta, P3];[Form factor, Delta, PO4];[Form factor, Delta, P6];[Form factor, Delta, C1]. According to Table 5, a trade-off between accuracy and lower computational complexity for real-time implementation of the proposed tremor detection algorithm led us to choose 3 s segments as the optimal length.

**Table 5:** Comparison between different window lengths for tremor prediction based on EEG data processing

Feature set		Classifier	Accuracy (%)	Sensitivity			Specificity			AUC
Feature set No.1	3-s	KNN	73.67 ± 9.56	70.90 ± 14.89	87.18 ± 10.00	61.52 ± 14.53	90.54 ± 8.92	92.50 ± 6.10	79.4 ± 9.30	0.74 ± 0.09
	5-s	Nave Bayes	73.82 ± 18.78	78.61 ± 24.47	88.45 ± 15.04	69.90 ± 29.16	92.80 ± 10.45	85.58 ± 14.05	86.00 ± 13.36	0.75 ± 0.18
Feature set No.2	3-s	KNN	81.29 ± 9.19	79.15 ± 11.38	84.86 ± 11.53	83.94 ± 20.93	90.37 ± 10.33	95.62 ± 6.29	86.57 ± 4.50	0.81 ± 0.09
	5-s	Nave Bayes	66.70 ± 17.00	66.58 ± 27.93	74.96 ± 25.17	65.39 ± 21.25	90.86 ± 10.72	85.69 ± 13.97	75.02 ± 19.88	0.67 ± 0.17

### Effect of PD symptom dominant side on the obtained results

Studies showed that left-dominant and right-dominant PD patients exhibited different electrical activity in the basal ganglia [40]. Additionally, structural deteriorations in PD, for example, the reduced fiber integrity in the nigrostriatal pathway are associated with the more affected side [41]. This might influence the observed EEG pattern for left and right-dominant PD patients. However, it is unclear how the differences in electrical activity or structure of the brain for left or right-dominant PD patients affect the observed EEG time-series at the scalp. To investigate the sensitivity of the results in the current study to dominant side, selected features that achieved the best classification accuracy (Feature set No.1) were compared between left and right-dominant PD patients. The results for different brain dynamics (i.e. eyes open, eyes closed, ON medication or OFF-medication) had been reported in Table 6. It is important to note that the sample size was different for each category, as it was not possible for some patients to detect tremor during some conditions due to either the absence of tremor or the tremor onset was outside 1-minute EEG recording span.

**Table 6:** P-values for differences between discriminative features (Feature set No.1) among left-dominant and right-dominant PD groups. Significant level was adjusted to 0.05 (LD: left-dominant, RD: right-dominant)

Feature	OFF-medication, eyes-closed (RD = 8, LD = 12)	OFF-medication, eyes-open (RD = 9, LD = 7)	ON-medication, eyes-closed (RD = 10, LD = 7)	ON-medication, eyes-open (RD = 11, LD = 7)
Form factor, delta, P4	0.79	0.6	0.06	0.05
Peak frequency, Gamma, FC6	0.34	0.34	0.09	0.05
Form factor, Alpha, CP2	0.31	0.56	0.15	0.08
HOS5, Gamma, F3	0.94	0.93	0.35	0.19
Skewness, Gamma, F3	0.77	0.8	0.34	0.18
Kurtosis, Gamma, FC6	0.27	0.36	0.79	0.91
HOS6, Gamma, FC6	0.95	0.99	0.36	0.22
L-skewness, Gamma, F7	0.72	0.88	0.17	0.08
L-kurtosis, Gamma, C4	0.24	0.22	0.42	0.33

### Comparison with other state-of-the-art methodologies

In this study, for the first time, the potential of EEG time-series for predicting PD tremor was evaluated. Our proposed methodology achieved an accuracy rate of 73.67% in tremor prediction. While this might be lower compared to other methods (see Table 7), most of these methods require the tremors to be initiated, whereas our proposed method was a predictive methodology that anticipated tremors before the initiation. Furthermore, against invasive DBS-based predictive systems, our proposed methodology is non-invasive and there are no concerns for physical damage to participants.

**Table 7:** Comparison between the proposed methods and other methods

Reference	Method	Features	Performance (%)
**This work**	EEG data analyses	Time and frequency domain features	73.67 (accuracy)
[42]	IMU placed on hand and analyzing data using deep neural network	Automatically learn features about data	97 (accuracy)
[43]	Wrist-worn 3D accelerometers and Deep learning: Convolutional neural networks	Non-negative factorization of frequency features	95
[31]	Bi-axial gyroscope data analyzed by adaptive Kalman filter and a wavelet transform	Spectral-temporal features	95.63
[44]	Features extracted from LFP from the subthalamic nucleus	Power in frequency bands	78 (accuracy)
[15]	Inertial sensors (accelerometer and gyroscope) attached to the index finger and wrist and SVM classifier	Root mean square, average peak power, standard deviation	88.9 (accuracy)
[45]	Accelerometer and gyroscope and bagged ensemble of decision trees	Sum of absolute differences and sums of squared magnitudes of accelerometer data	82
[17]	Surface electromyogram and acceleration signals	Power at peak frequency, energy of selected wavelet coefficients, Shannon entropy, recurrence quantification parameters	80.2 (accuracy)
[46]	Accelerometers and surface electromyography placed on forearm and shank and dynamic neural network algorithms	Evolving temporal characteristics (energy, autocorrelation)	94.9 (sensitivity) 97.1 (specificity)
[21]	Local field potentials obtained from a DBS system	Energy, variance, zero crossing rate, autocorrelation, information theory, power spectral density magnitudes	86 (accuracy)
[22]	Radial basis function neural network and particle swarm optimization technique based on local field potentials	Frequency changes between pre-tremor and tremor conditions	89.91 (accuracy)
[16]	Miniature gyroscopes placed on forearm	Hilbert transform and instantaneous frequency	99.5 (sensitivity) 94.2 (specificity)

## Discussion

In this study, the potential of EEG time-series was evaluated for the prediction of PD tremors. Before this, only a limited number of studies proposed methodologies for tremor onset prediction using invasive modalities such as deep brain electrode insertion [22]. According to Table 3, the discriminative feature in the low-frequency region of EEG (delta, theta, and alpha) was mainly the form factor. This result indicated that the overall complexity of the low-frequency content of EEG increased significantly before tremor initiation. Furthermore, the results indicated that statistical features in the gamma band significantly increased before tremor initiation. However, our analysis did not show any observed trend for discriminative features during time evolution before tremor onset. According to the literature, different neural circuits including basal ganglia and cerebello-thalamo-cortical circuits are engaged in resting tremors [47]. Considering the dimmer-switch model, a successive activity starting from the internal pallidal globus in basal ganglia, propagates to the cerebello-thalamo-cortical and then to the thalamus and cerebellum [48]. This cascade of activities is observed in different periods of EEG time-series and not concentrated in a limited duration segment. This might be the reason that no observable trend was seen for the change of feature values before tremor initiation.

According to Table 5, increasing the analysis window size did not enhance the prediction accuracy significantly (note the higher standard deviation for 10-fold cross-validation results for 5 s windows) and also increased the computational complexity. In this regard, for EEG feature extraction from pre-tremor, tremor, and non-tremor events, 3-s windowed segments were used. The reason for such selection was that for a shorter length window, the calculation of features such as sample entropy or features extracted from the power spectrum might not be reliable. Furthermore, for a longer window length, the computational and time complexity might increase that affected the real-time feasibility of the algorithm.

The tremor prediction ability for PD patients is useful since it can reduce the number of stimulations in DBS or electrical or magnetic stimulations of nerves and muscles to disrupt the tremor signals [6, 7, 49] and hence save the battery power [22]. The results of this preliminary study showed that a combination of time and frequency domain extracted features from EEG time-series of limited numbers of EEG channels could be considered as a useful strategy for predicting tremor onset. According to Table 4, a KNN classifier obtained a predicting accuracy of 73.67 ± 9.56% and the area under ROC curve of 0.74 ± 0.09 for the pre-tremor recognition. This result was obtained for six EEG recording locations (P4, FC6, CP2, F3, F7, and C4). Reducing the feature set using NCA even increased classification accuracy to 81.29 ± 9.19% and area under ROC curve to 0.81 ± 0.09. However, the detection accuracy using EEG biomarkers was smaller than the accuracy obtained by local field potentials (89.91%) [22]. Surprisingly, the main locations in which pre-tremor condition was distinguished from tremor or non-tremor conditions consisted of motor function area in the right brain lobe (FC6, C4, CP2 and P4), and the left dorsolateral prefrontal cortex (F3, F7). Previous studies highlighted the activation of dorsolateral prefrontal cortex in self-initiated movements [50]. Furthermore, the posterior parietal cortex contains neural pathways for voluntary hand movements [51]. Studies suggested that gamma oscillatory activity increased near the triggering of movement [52]. In this regard, increasing gamma content in the movement control regions of the brain (prefrontal and centroparietal parts as specified by F3/F7 and FC6/C4/CP2/P4 EEG channels, respectively) before tremor initiation can be justified. In future studies, the discriminative capability of prefrontal and centroparietal areas for tremor prediction should be studied more precisely. The results reported in Table 6 also showed that there were no significant differences between discriminative features for right-dominant and left-dominant PD patients (p > 0.05).

## How to use the proposed methodology in a real application?

The proposed methodology can be implemented in a system consisting of an EEG recorder, an accelerometer, and a controller. For an individual participant, according to the results of this study, EEG data should be recorded from selected channels (F3/F7/P4/C4/CP2/PC6). Features according to the proposed discriminative features should be calculated for consecutive, overlapped 3-s EEG time-series. For characterizing, “Tremor” and “Non-tremor” conditions, a trained observer can label data by an external trigger (for example by pressing a button, mouse keys or a key on computer keyboards when hand tremor event observed) based on visual inspection. This can be repeated for a pre-specified training duration. A simple code can refine the pure tremor and non-tremor windows by analyzing accelerometer data, tremor start and end time. Furthermore, by analyzing accelerometer data, a customized code can easily label “Pre-tremor” conditions. For several iterations, the feature space for “Tremor”, “Pre-tremor” and “Control” conditions is constructed. According to these labeled data, a classifier is trained. In the real situation, when most discriminant EEG channels, frequency bands and features were considered and calculated for ongoing EEG time-series, the classifier determines to which class the current condition belongs. According to the classifier output, in the case of the Pre-tremor condition, a stimulus for tremor suppression can be applied. The decision can be rechecked by accelerometer data analysis. In the case of an incorrect decision (false alarm for tremor initiation or missed tremor events), the applied stimulation (for example electrical or magnetic stimulation) can be stopped. It should be noted that the purpose of integrating EEG data and accelerometer data analysis was to increase the chance of tremor suppression before its initiation. Since the tremor characteristics may be different between PD patients, it is suggested to train the predictor for each participant.

## Study limitations and future works

Tremor in PD patients is a very complicated phenomenon. Considerable clinical heterogeneities were observed for PD tremors [53] in a way that the characteristics of tremor such as amplitude, frequency, and inter-tremor intervals are completely different between PD patients. In order to limit the possible biases in the obtained results due to inherent inter-subject or intra-subject heterogeneities, long recorded data analysis for tremor is needed. Unfortunately, the available data for the current study consisted of very short recordings (1 min for eyes-open or eyes-closed resting-state conditions). Such short length data contained a limited number of tremors. This limitation precluded a through exploration of intra-subject variability in measures derived from EEG time-series before tremor onset. Furthermore, tremors are highly coupled with brain dynamics. For example, cognitive stress or the level of hormones affects significantly tremor characteristics [53]. In the current study, only two specific brain dynamics, i.e. eyes-open and eyes-closed conditions were considered; however, in real life, subjects may be involved with various brain processing activities, including problem-solving, visuospatial processing, or auditory tasks. This is the next limitation of the current study in which the obtained results may be restricted to only brain resting-state conditions. In fact, in the current study, only the potential of EEG time-series for tremor anticipation was evaluated, and the results were promising. In other words, this study should be considered preliminary. To fully assess the clinical significance of these findings, longer tremor data and EEG recordings during various brain activities are needed. In addition, using EEG recording units (electrodes and an amplifier unit) may be considered inconvenient for a long time. However, the use of a non-invasive EEG set is probably more favorable than invasive deep-brain implanted electrodes for PD people with tremors. Increasing the prediction potential of the current work using more advanced EEG signal processing strategies such as adaptive signal decomposition and processing strategies [54, 55] should be considered for future.

## Conclusion

Reducing the power consumption of the stimulating device and mitigating the side effects are among the most important advantages of tremor prediction. In the current study, the potential of EEG for tremor prediction was investigated. Results showed that EEG might be a useful tool; however, due to the complexity of tremor events, and unknown relationships between different brain dynamics and tremor characteristics, it is necessary to check the proposed methodology for tremor prediction in a wide range of brain dynamics. Furthermore, a longer dataset for considering heterogeneous and intra-subject variability characteristics of tremor is demanded for future studies.

## Data Availability

The EEG data for this manuscript can be found at http://predict.cs.unm.edu/downloads.php under Parkinson’s Rests project. This data was released and freely available according to James F. Cavanagh research.
